# Electric tuning of direct-indirect optical transitions in silicon

**DOI:** 10.1038/srep06950

**Published:** 2014-11-07

**Authors:** J. Noborisaka, K. Nishiguchi, A. Fujiwara

**Affiliations:** 1 NTT Basic Research Laboratories, NTT Corporation, 3-1 Morinosato Wakamiya, Atsugi, Kanagawa, 243-0198 Japan

## Abstract

Electronic band structures in semiconductors are uniquely determined by the constituent elements of the lattice. For example, bulk silicon has an indirect bandgap and it prohibits efficient light emission. Here we report the electrical tuning of the direct/indirect band optical transition in an ultrathin silicon-on-insulator (SOI) gated metal-oxide-semiconductor (MOS) light-emitting diode. A special Si/SiO_2_ interface formed by high-temperature annealing that shows stronger valley coupling enables us to observe phononless direct optical transition. Furthermore, by controlling the gate field, its strength can be electrically tuned to 16 times that of the indirect transition, which is nearly 800 times larger than the weak direct transition in bulk silicon. These results will therefore assist the development of both complementary MOS (CMOS)-compatible silicon photonics and the emerging “valleytronics” based on the control of the valley degree of freedom.

Bulk silicon has an indirect band gap and a multiple degenerate valley structure in the conduction band. Electrons occupy the minimum energy states with the crystal momentum far from zero (near the X-point) and holes occupy the maximum energy states in the valence band at zero crystal momentum (Γ-point) (Fig. 1(a)). Since photons do not carry significant momentum and a dipole transition requires momentum conservation, an optical direct transition is not allowed in bulk silicon and a weak phonon-mediated indirect transition is dominant. To overcome this inherent constraint, many attempts have been made to improve emission efficiency by using quantum confinement^1^,^2^,^3^,^4^,^5^,^6^, doping^7^,^8^, strain^7^,^9^, and heterogeneous technology^4^,^10^,^11^. However, most of these approaches rely strongly on a specific fabrication process, and the origin of the improvement is not fully understood due to the lack of tunable physical parameters.

Recently, “valleytoronics”, which utilize valley degrees of freedom to create new functions, have been attracting much attention^12^,^13^,^14^,^15^,^16^. Valley splitting in a silicon has been widely studied two dimensional electron systems (2DESs)^17^,^18^,^19^,^20^,^21^,^22^,^23^,^24^,^25^,^26^,^27^,^28^,^29^,^30^,^31^, quantum dots (QDs)^32^,^33^ and dopants^34^,^35^. It is known that the physical origin of the valley splitting results from the coupling of valley generated states due to the breakdown of the translational symmetry and the resultant momentum dispersion of electrons. The energy of the valley splitting at both a conventional silicon metal-oxide-semiconductor (MOS) and a Si/SiGe interface is typically several hundred microelectron volts^20^,^21^,^22^,^23^,^24^,^25^,^26^,^27^,^28^,^29^, and its magnitude can be tuned by controlling the gate electric field. Recently, an anomalously large valley splitting of a few tens of millielectron volts has been reported using a specially prepared Si/SiO_2_ interface formed on SIMOX (separation by implantation of oxygen) silicon on an insulator (SOI) substrate and a large gate electric field^17^,^18^, suggesting a large momentum dispersion of the conductive electrons. Since such a large energy of the valley splitting is comparable to that of dopant impurities, we expect that these electrons can be directly dipole–coupled to holes, thereby emitting photons efficiently. However, it is not easy for such electrons to couple with holes at the interface since the large electric field makes them separate in real space. In this study we try to solve this problem by using thin silicon quantum wells (QWs) to confine both electrons and holes so that their spatial overlap is significant even in a high field, thus making it possible to maintain a large overlap in both momentum and real space.

In the present work, we form a thin silicon gated p-i-n MOS diode on a SIMOX wafer (Fig. 1(b)). The front-gate oxide (FOX) is formed by conventional thermal oxidation. The main difference between our devices and typical ultra-thin MOSFETs is the use of buried oxide (BOX), which is formed by employing high temperature thermal treatment followed by oxygen ion implantation. This treatment causes large valley splitting when electrons distribute at the BOX interface. The width and length of the gate is 200 and 400 μm, respectively. The thicknesses of the QWs are 4.3 and 6.0 nm. (Further details about device fabrication are provided in the Method section.) We find that the silicon QW diode exhibits strong and electrically-tuned direct optical transition, which would assist the development of CMOS-compatible silicon photonics^36^,^37^,^38^.

## Results

**Figure 2:**
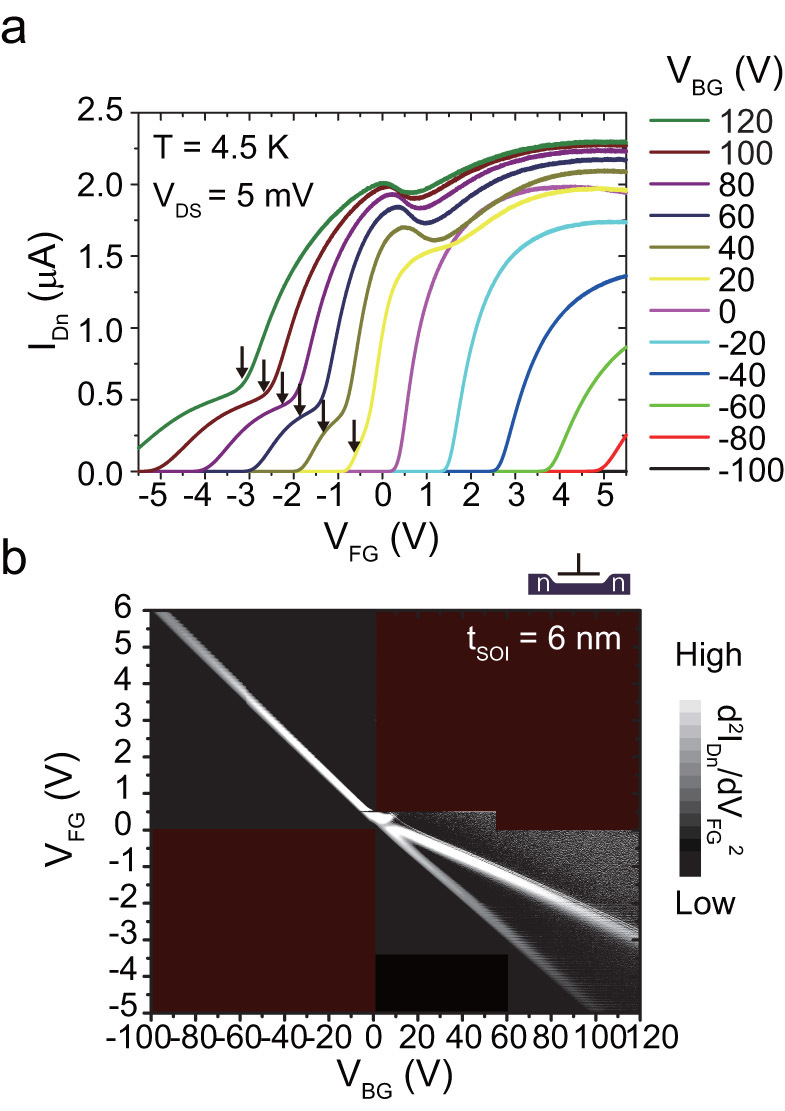
Transport measurements of valley splitting. (a) Drain current I_Dn_ through an electron channel as a function of V_FG_ at T = 4.5 K for t_SOI_ = 6 nm. In this measurement, only n-type contacts are used and constant drain voltage V_D_ of 5 mV is applied. The arrows indicate the onset of current conduction though the valley antibonding state, which corresponds to the bright upper line for V_BG_ > 0 in Fig. 2(b). The second dip (without arrows) is caused by the onset of current conduction through the other (front-gate) side channel. (b) The doubly differentiated drain current with respect to V_FG_. The axes are adjusted to fit the diagonal line on the threshold of current conduction taking into account the size of the BG and FG capacitance. For a positive V_BG_ value, the onset of conduction appears as a bright diagonal line that deviates slightly from the expected line because of an artifact caused by taking the second derivative. Another bright line, which corresponds to the arrows in Fig. 2(a), for a positive V_BG_ value arises from the level coincidence between the source Fermi surface and the antibonding state.

First, we investigated valley splitting under unipolar conditions where only n+ contacts are used to measure the electron conduction, with reference to a previous experiment^17^,^18^. Figure 2 shows the drain current (I_Dn_) and its second derivative (∂^2^I_Dn_/∂V_FG_^2^) as a function of V_FG_ and V_BG_. The bright diagonal line in the ∂^2^I_Dn_/∂V_FG_^2^ panel indicates the onset of current conduction through electron ground states in the QW. For a positive V_BG_ value, another line structure appears at a different angle; the structure was previously studied in detail including the Shubnikov-de Haas oscillation of 2DESs under a magnetic field^17^,^18^,^30^. From the appearance of periodic Shubnikov-de Haas oscillation and the valley degeneracy estimated by both the carrier density and two dimensional density of state, it was identified as a valley excited state arising from anomalous large valley splitting at the Si/BOX SiO_2_ interface. The ground and excited states are the orbital bonding and antibonding states, respectively, originating from the coupling of the two [001] valley states. The onset of the occupation of the excited states modifies the electron mobility^31^, which induces a structure in the I_Dn_ curves (Fig. 2(a)). The estimated energy of the valley splitting is 21 meV at V_BG_ = 60 and V_FG_ = −1.6 V where the electron density is approximately 1.7 × 10^12^ cm^−2^. The splitting at a normal (front) Si/SiO_2_ interface is at least one order of magnitude smaller and is undetectable for a negative V_BG_ value due to the inhomogeneous broadening of the conduction onset voltage. We should mention here that the physical origin of the large valley splitting remains unclear, and is still under debate although there are several proposals regarding the origin such as interface states at Si/SiO_2_^39^, roughness^27^ or disorder^40^.

**Figure 3:**
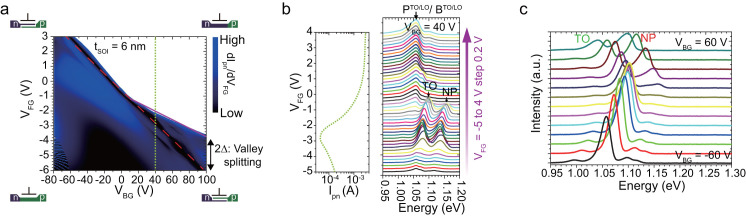
Transport measurements and EL spectra under bipolar operation. (a) The first derivative of current through n- and p-type contacts with respect to V_FG_ for t_SOI_ = 6 nm. Constant diode bias for n- and p-type contacts of V_Sn_ = −1.5 V and V_Sp_ = 2.5 V are applied, respectively. Carrier density and valley splitting are adjusted by using the front and back gates. The broken red and solid purple lines indicate the current minima and provide an eye guide for the antibonding state of the valley coupled states, respectively. (b) Injection current through n- and p-type contacts, I_pn_, as a function of front-gate voltage V_FG_ at V_BG_ = 40 V. Simultaneously obtained EL spectra are also shown on the right, where the integration time of each spectrum is 10 s. (c) EL spectra given by the bias conditions slightly shifted from the broken red line in Fig. 3(a). The energy differences between the NP and TO peaks at a given V_BG_ value are consistent with the TO phonon energy in silicon (~58 meV).

We then tested diode operation with n+ and p+ contacts and found that the diode current also has a valley-related signature (its derivative is shown in Fig. 3(a)). The onset of valley splitting is shifted slightly toward negative V_FG_ and V_BG_ values due to the bias applied to the n+ contact, but the features in Fig. 2(b) are consistently reproduced (See also Supplementary Information Sec. II-A). The appearance of valley splitting in I_pn_ indicates that electron mobility still has a significant effect on the bipolar current. The electroluminescence (EL) spectra taken along with the constant V_BG_ value in Fig. 3(a) are plotted in Fig. 3(b). The spectra are mainly dominated by three features arising from the origin of their recombination. For a high negative or positive V_FG_ value, an impurity recombination accompanied by transverse or longitudinal optical (TO/LO) phonons (P^TO/LO^ or B^TO/LO^) (~1.06 eV) at heavily doped contacts dominates the spectra^41^,^42^. For a specific V_FG_ value, two other peaks develop. When we take these peak energies with an energy separation of ~59 meV^43^,^44^,^45^ and the rearrangement of ground states by quantum confinement into consideration, these two peaks are assigned as a transverse optical (TO) phonon mediated free-exciton (I^TO^) emission and a non-phonon (NP) free-exciton (I^NP^) emission, respectively. For these gate-biases, electrons and holes recombine within an undoped QW and their EL intensity maintains a high value although I_pn_ is rather small. To focus on the light emission from the undoped QW channel, the EL spectra taken along the current minima, which correspond to the broken red line in Fig. 3(a), are plotted in Fig. 3(c). There is a noticeable change in the EL spectra from a negative to positive V_BG_ value; the peak intensity of the NP emission is small for a negative V_BG_ but it gradually increases from almost zero to a large positive value. The intensity of the NP peak strongly depends on |V_BG_| and develops greatly with a positive V_BG_ value. Since large valley splitting appears for a positive V_BG_ value and its magnitude increases as V_BG_ increases, these results clearly indicate that a direct-indirect optical transition can be tuned by changing the gate electric field and the tunability strongly correlates with the magnitude of the valley splitting. Although there can be several possible origins for a strong NP transition such as interface states that may exist at the special interface, which will be discussed later in more detail, we believe that one probable origin of this strong NP transition is the valley-coupled state we observed in the conduction measurement, which exhibits a large valley splitting energy as impurity states do.

**Figure 4:**
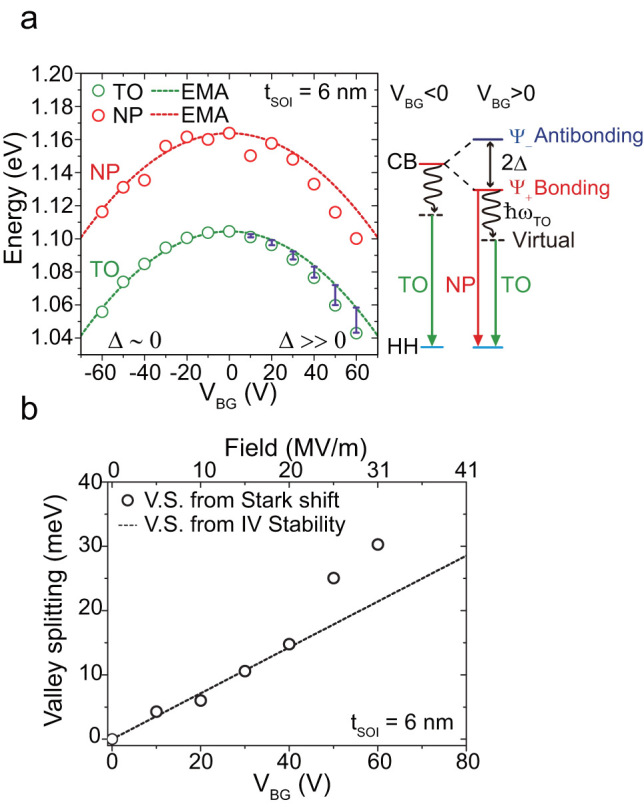
Verification of valley splitting estimated from optical and electrical measurements. (a) The peak energy variation as a function of V_BG_. The dotted lines are the Stark shift given by EMA calculation of t_SOI_ = 6 nm. Since the valley splitting for a negative V_BG_ value is negligible, we directly estimate the valley splitting from the energy difference between negative and positive V_BG_ values. Corresponding emission line spectra are shown on the right. (b) The valley splitting derived from the Stark shift is plotted with that obtained from the current-voltage stability diagram. Open black circles indicate the Stark shift of the TO peaks for t_SOI_ = 6 nm. The slight deviations may be ascribed to the difference in the electric field caused by the bipolar condition or the ambiguity of the peak positions.

Figure 4(a) shows the TO and NP peak energies as a function of V_BG_. The energies show an almost quadratic dependence, indicating the occurrence of a quantum confined Stark shift (Supplementary Information Sec. II-C). The observation of the quantum confined Stark shift indicates that the emission peaks are related to the confined state in the QW. The energy of the Stark shift is calculated using a single-valley effective mass approximation and plotted as a dotted line in Fig. 4(a). It should be noted that there is slight asymmetry for V_BG_. We believe that this asymmetry is a result of the large valley splitting for a positive V_BG_. Since valley splitting for V_BG_ < 0 is negligible, we can fit the energy differences as δE = αV_BG_^2^, where α is a constant. In contrast, for V_BG_ > 0 the valley splitting is significant. Thus it is fitted by δE = αV_BG_^2^-Δ and we estimate the valley splitting energy, 2Δ. In Fig. 4(b), the valley splitting estimated from these EL peak positions is plotted with that estimated from electrically measured data (Fig. 2(b)). They show fairly good agreement, thereby supporting our interpretation that NP transitions are mediated by the ground state of valley coupled states. Additionally, we can estimate the electric field in a QW and splitting coefficient^27^,^26^ γ (unit in Coulomb·m) supposing a linear relation of 2Δ = γ *F*, where Δ is half of the valley splitting in electron volts and *F* is the effective field for the electrons. Coefficient γ is approximately 10^−9^ for BOX interfaces (Supplementary information Sec. II-C). Meanwhile, it is 2.6 × 10^−11^ for the standard Si/SiO_2_ interface in bulk MOSFETs^19^,^20^,^21^,^23^.

**Figure 5:**
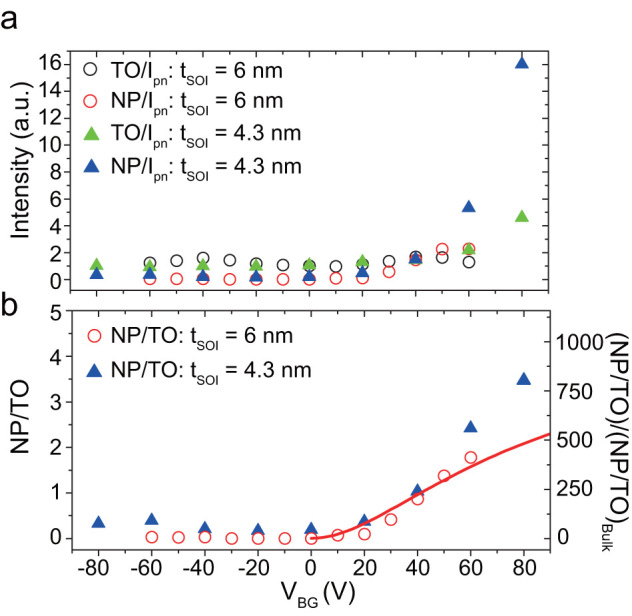
NP emission versus valley splitting. (a) Products of integrated EL intensity divided by injection current at a given V_BG_ value. They are plotted with values normalized by that for V_BG_ = 0 V for t_SOI_ = 6 nm, which is defined as the "bulk limit" because the valley splitting is small under this condition. (b) Non-phonon (NP) and transverse optical phonon (TO) ratio (I_NP_/I_TO_) for t_SOI_ = 6 nm and t_SOI_ = 4.3 nm. The left ordinate is the raw I_NP_/I_TO_ ratio, and the right one is that divided by I_NP_/I_TO_ at the bulk limit (V_BG_ = 0 V and t_SOI_ = 6 nm). The solid red line corresponds to the I_NP_/I_TO_ intensity estimated by numerical calculation (for t_SOI_ = 6 nm) based on the EM framework described in the main text.

To discuss the enhancement of the direct optical transition qualitatively, we plot the integrated EL intensities divided by the injection current in Fig. 5(a). The NP intensities increase with increasing positive V_BG_ value and its intensity in a thinner QW is higher than that in a thicker one. Since the increase is observed only for a positive V_BG_ value, we consider it to be a consequence of the large valley coupling. The larger enhancement in a thinner QW is probably due to the stronger confinement. At V_BG_ = 80 V, the efficiency of a NP direct transition was 16 times greater than that of a bulk indirect TO transition. In Fig. 5(b), intensity ratio I^NP^/I^TO^ is plotted for a given V_BG_ value. The I^NP^/I^TO^ ratio increased by a factor of 4 at V_BG_ = 80 V, indicating that NP transition is now the dominant recombination, where the direct NP transition rate is 800 times greater than that under a bulk condition. We here compare our results with impurity-based luminescence in silicon. Bi in silicon has an activation energy of 71 meV^46^ and the energy spitting between the ground 1s (A_1_) and the excited 1s (T_2_) state is 39 meV^46^. In Bi doped silicon, the dominant radiative process is recombination through the exciton bound at neutralized donor Bi^0^X and its NP to TO ratio Bi^0^X^NP^/Bi^0^X^TO^ reaches two^44^,^47^, which is comparable to the value in our experiment.

To explain our experimental results semi-quantitatively based on the 2DES model, we calculated the NP emission rate based on an electric breakthrough model derived from an extended zone effective mass (EM) theory^19^,^20^,^21^. It is known that this EM model provides similar results to other theories^25^,^26^,^27^,^29^. The electric field dependence of valley splitting in the model is explained by the following 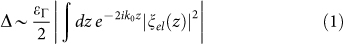
where ε_Γ_ is the energy difference between the Γ_15_ and Γ_1_^u^ states, k_0_ is a wavevector for conduction minima, and *ξ_el_(z)* is an electron envelope function. This equation well reproduces the valley splitting observed in standard MOSFETs and its linear dependence on the electric field (Supplementary Information Sec. II-D). If we use eq. (1) for a silicon QW, the valley splitting (2Δ) is estimated to be 0.77 meV at *F_SOI_* = 30 MV/m for t_SOI_ = 6.0 nm. Although this EM model cannot explain the anomalously large valley splitting in our QW, we believe that it is still meaningful to use the EM framework to calculate the optical transition probability for a discussion of the electric field dependence because the large valley splitting is also proportional to the electric field.

The NP intensity in this model combined with the envelope function approximation^48^,^49^ is given by 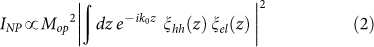
where Μ_OP_ is a momentum matrix element of the Bloch function |*<u_hh,k_|p_z_|u_el,k_>*| due to a perturbation caused by a vacuum field and *ξ_hh_(z)* is a hole envelope function. The M_OP_ element is composed of the <Γ_25_|Γ_15_|Γ_15_+Γ_1_^u^ (+Γ_1_^l^)> symmetry^50^, which has a non-zero component, reflecting the fact that an optical transition is allowed at the Γ point. Since the dominant term of M_OP_ is <Γ_25_^l^|p|Γ_15_> and its variation caused by the gate field is at most 30% (Supplementary Information Sec. II-D), it is set at a constant value for simplicity. The second part of eq. (2) extracts the k_0_ component of the Fourier coefficient for a multiple product of electron and hole envelope functions and is a function of the gate field. This part is closely related to the valley splitting described by eq. (1) because it extracts its 2k_0_ component as the squared product of the electron envelope function. In a usual envelope function (without singularity), the amplitude of the k_0_ component in regard to the Fourier coefficient follows that of 2k_0_. Accordingly, if the QW is sufficiently thin, eq. (2) correlates closely with eq. (1). Therefore, we can expect that the NP transition increases according to the increase in the valley splitting.

The intensity of an indirect TO phonon mediated transition (I_TO_) is proportional to the square of the overlap integral of the electron and hole envelope functions 
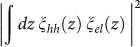
. Therefore, the intensity ratio I_NP_/I_TO_ is given by 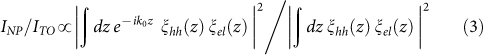
The field dependence of eq. (3) is plotted as a line in Fig. 5(b), where we subtract a constant offset to compensate for the enlarged zero-field valley splitting (residual product that appears due to the introduction of the proportional factor in eq.(2), see also Supplementary Information Sec. II-D). Although our 2DES model might be rather phenomenological due to a lack of the identification in regard to the large valley coupling, the tendency of I_NP_/I_TO_ agrees reasonably well with the experiment. Further application of the field finally results in a decrease in the I_NP_/I_TO_ ratio due to the separation of the envelope functions but it might be possible to overcome this by using much thinner QWs (Supplementary Information Fig. S6).

## Discussion

We discuss here another possible origin of the observed strong NP transition such as quantum dots (QDs) formed by the interface roughness and interface states.

First we deduced the QD model as a cause of strong NP transitions. The roughness of the BOX/Si interface is much higher than that for FOX/Si, whose potential variation could localize electrons and thereby form QDs at the BOX/Si interface for positive V_BG_ values. These QDs may cause strong NP transitions owing to the carrier confinement. In this case, however, a strong NP transition should also be observed for negative V_BG_ values because the holes can also be localized by potential variations, the absence of such behaviour in the experiment suggests a low probability for this cause.

Electrons trapped at the interface states represent another possible origin, where the strong light emission is attributed to the interface states^51^. The reported emission energy is approximately 1.6 eV and is not similar to our case. Though in our case the interface states, whose energy is similar to that of the confined electron state in the QW, may play a role because the emission energies follow the quantum confined Stark shift. Further study will be needed to address the cause of strong NP transitions at the BOX/Si interface using other methods such as C-V measurement^39^.

If our conductive-electron model is correct, the question is what causes the large valley coupling. It is not clear as noted before, but it would be reasonable to speculate that atomic-scale scattering centers located at the interface, which may be formed by interface imperfection or strain, are the cause. Their rapidly varying potential significantly changes the electron wavenumber and causes the large valley splitting.

We have shown the enhancement of a NP optical transition by using strong valley coupling and shown its electric field dependence in silicon. The NP direct optical transition intensity can be electrically tuned to make it three orders of magnitude greater than under a bulk condition. A simple model based on the effective-mass theory can explain the results qualitatively, and the model highlights the strong correlation between the valley coupling and the direct optical transition.We believe that our findings will open up the way to the use of silicon as a light emitting material as well as help us to explore new physics and applications involving the control of valley states.

## Methods

### Device fabrication

The SOI-MOSFETs used in the experiments were fabricated on SIMOX (separation by implantation of oxygen) (001) wafer^52^ annealed at 1350°C for 40 hours to minimize the influence of interfacial roughness at the Si and buried oxide (BOX) interface^53^,^54^. The SOI layer was then thinned by using thermal oxidation and etching with dilute hydrofluoric acid solution. This was followed by dry gate oxidation at 700°C and etching to define the device geometry. Two SOI thicknesses (t_SOI_), nominally 4.3 and 6 nm (measured by ellipsometry), were prepared. Front poly-Si gates were then formed to define the channel width and length. Then heavily doped n- and p-type contacts were formed by phosphorous and boron implantation. Finally, the devices were annealed in a hydrogen atmosphere to activate the dopants. The nominal thicknesses of the front-gate oxide (t_FOX_) and the buried oxide (t_BOX_) were approximately 20 and 400 nm, respectively.

## Author Contributions

J.N. designed and planned the experiments, fabricated the devices, and collected and analyzed the data. K.N. supported the device fabrication. A.F. planned and supervised the study. J.N. and A.F. wrote the manuscript. All authors discussed the results and commented on the manuscript.

## Supplementary Material

Supplementary Information Supplementary information

## Figures and Tables

**Figure 1:**
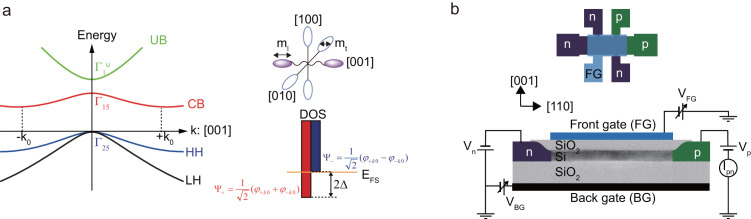
Device design. (a) Energy dispersion of conduction band (CB), heavy hole (HH), light hole (LH) and upper band (UB) along [001]. In quantum well, two-fold degenerate valleys take ground states and the residual four-fold degenerate valleys are lifted. For strong confinement conditions, the two-fold degenerate valleys couple and form bonding and antibonding states because of the significant momentum dispersion. Even for finite valley coupling, the main components of the envelope function in momentum space stay around +k_0_ and -k_0_. The top right schematic illustrates the equi-energy surface of conduction minima states, where m_l_ and m_t_ are the longitudinal and transverse effective mass of the conduction electrons, respectively. Valley splitting 2Δ is defined in the bottom right schematic, where the Fermi surface of contact is shown as an orange line that aligns with the antibonding state (fully valley polarized). (b) Device cross-section and experimental configuration with TEM image of SIMOX gated p-i-n MOS diode. The device has n- and p-type doped contacts. These contacts are used to inject electrons (n-type) or holes (p-type) into an undoped SOI channel. The distribution of the injected carriers is controlled by the front gate (FG) and the back gate (BG).
