# Supplementary material for: Electric tuning of direct-indirect optical transitions in silicon
Source: Sci Rep. 2014 Nov 7;4:6950. doi: 10.1038/srep06950 (PMC4223641; doi:10.1038/srep06950)
Supplement: Supplementary Information [file srep06950-s1.pdf]

Supplementary information:
Electric tuning of direct-indirect optical transitions in silicon

J. Noborisaka*, K. Nishiguchi and A. Fujiwara

In this supplementary information, we describe device fabrication and the data analysis method. We also describe some valley related physics for a conventional Si/SiO_2 interface based on effective mass (EM) theory, where the valley splitting is an order of magnitude smaller than the results at a BOX/Si interface. Nevertheless, the underlying physics related to valley splitting and optical transition seems to be fairly well explained by the theory. We start with device fabrication and data analysis and finally turn to valley related physics including a field dependent direct optical transition.

I. Device fabrication

The devices are fabricated on SIMOX (separation by implantation of oxygen) (001) wafer [S1] annealed at 1350 °C for 40 hours to minimize the influence of interfacial roughness at the Si and buried oxide (BOX) [S2-S4]. In a previous work it was reported that this treatment results in the formation of lattice steps and terraces at the silicon/buried-oxide (BOX) [S3, S4] interface and the interfacial flatness improves within the terraces while it deteriorates over a long range of about 1 μm due to the steps, and the average period of the steps aligned with <110> was several 100 nm. When fabricating our device, after this thermal treatment, we thinned the silicon-on-insulator (SOI) layer by thermal oxidation and etching with dilute hydrofluoric acid solution. This was followed by dry gate oxidation at 700˚C to form the front SiO_2/Si interface and etching to define the device geometry. We prepared samples with two SOI thicknesses (t_{SOI}), nominally 3 and 6 nm (measured by ellipsometry). The front poly-Si gates were then formed to define a channel with a width (W) of 200 μm and a length (L) of 400 μm. The nominal thicknesses of the front-gate oxide (t_{OX}) and the buried oxide (t_{BOX}) were about 20 and 400 nm, respectively. After forming the poly-Si front gate, we formed the p^+ and n^+ contacts by the ion implantation of boron and phosphorous, respectively, where the SOI thickness of these contact regions was 50 nm. To activate implanted dopants, thermal treatment was performed at 1000 ˚C for 20 minutes in a hydrogen atmosphere. This thermal treatment sometime causes the diffusion of dopants from a poly-gate into the SOI layer for a thin gate oxide [S5] but it is negligible in the present case thanks to use of a thicker front gate oxide.

II. Data analysis

A. Valley related structure in Ipn

In the main text we plot the first derivative of I_{pn} with respect to V_{FG} in Fig. 3a. The second derivative is shown in Fig. S1. The figure shows a similar structure to the second derivative of I_{Dn} (Fig. 2b) and the line structure is attributed to valley splitting because of its splitting size. In the first derivative of I_{pn}, the threshold of the color plot is determined by the peak position of the second derivative. Thus, we identified that the line structure in Fig. 3a is caused by valley splitting.

B. EL peak assignments

The peaks were assigned by multi-peak Gaussian fitting. The EL spectrum for t_{SOI} = 6 nm at zero gate bias is decomposed into four peaks (P1: 1.04, P2: 1.10, P3: 1.14, P4: 1.16 eV) where the most intense peak is P2 as shown in Fig. S2 (a). With undoped Si at a low temperature, the dominant radiative recombination process is commonly caused by a TO phonon mediated

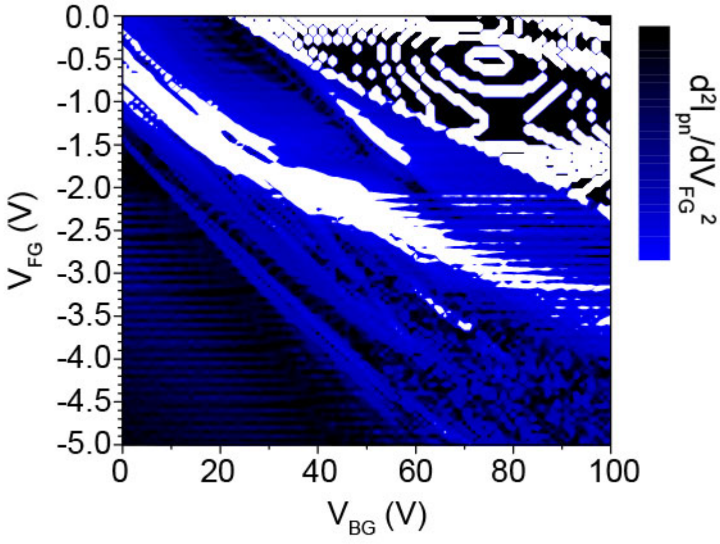

Figure S1 | Second derivative of I_{pn}. Second derivative of I_{pn} at the bias conditions for (V_n, V_p) = (-1.5, 2.5) V

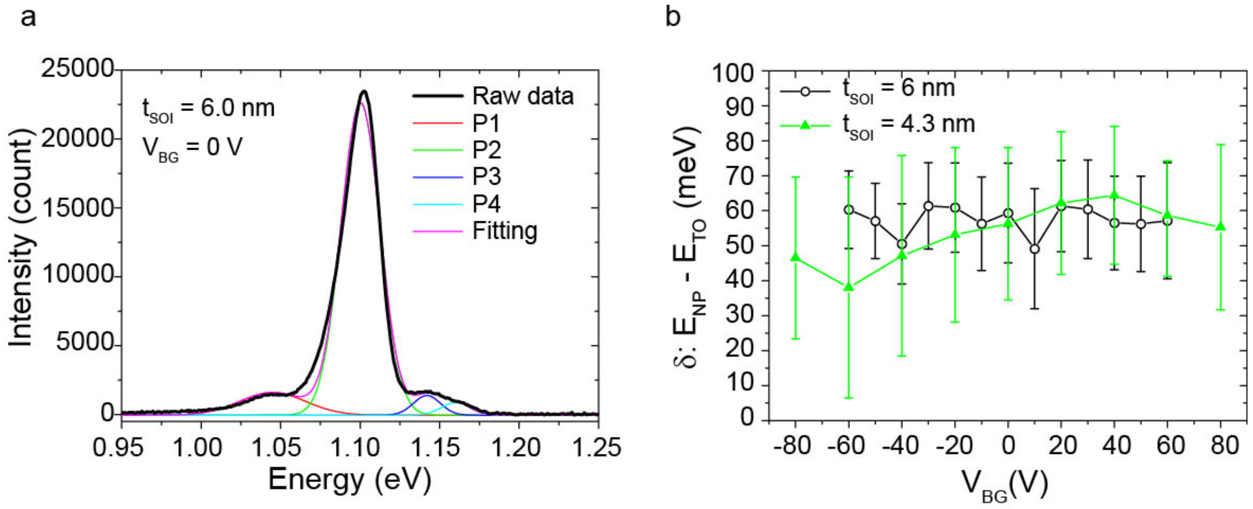

Figure S2 | EL spectrum analysis. a, Deconvolution of the EL spectrum taken at V_{BG} = 0. All NP and TO peaks are fitted by Gaussian distributions. **b,** Peak energy differences between NP and TO associated with V_{BG} variation. The symbols indicate the average energy difference δ between NP and TO. The bars cover the larger standard deviation between NP and TO at a given V_{BG}.

free-exciton transition with a typical peak energy of 1.097 eV [S6]. Therefore we attribute P2 to the TO peak. Although there is some variation in the energy difference (δ) between P2 (TO) and P4 (Fig. S2 (b)) probably due to error related to peak deconvolution, it is almost constant and close to the TO phonon energy, and so we conclude that P4 originates from a non-phonon (NP) peak. The other possibilities such as the emission related to the QW excited state can be excluded because δ shows significant V_{BG} dependence in theoretical calculations. Similarly P1 and P3 can be assigned to TO + O^{Γ} and a TA phonon mediated transition, respectively.

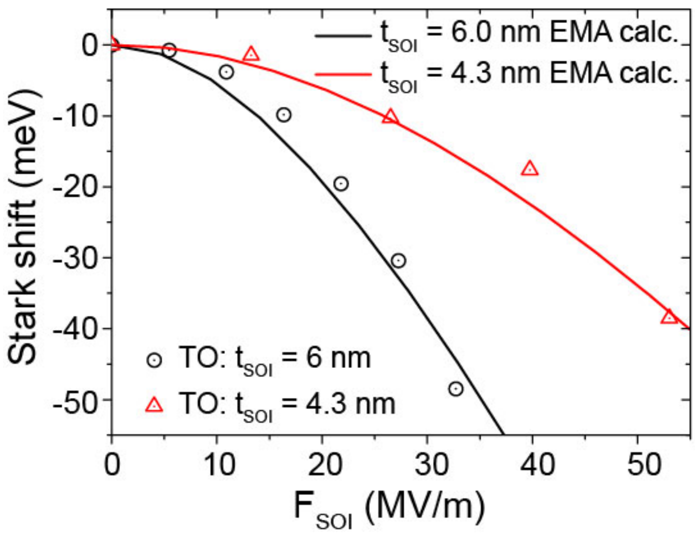

Figure S3 | Stark shift analysis. The symbols indicate experimental TO peaks at $V_{BG}<0$. The sold lines are Stark shifts calculated by EMA. A lever factor is obtained by comparing the quadratic fitting parameter for experimental data with that for EMA results.

C. Estimation of valley splitting energy from EL peaks

Because the emission peak energy should reflect the valley splitting energy as well as the energy shift due to the quantum confined Stark effect (QCSE)[S7], we can estimate the valley splitting energy from EL data, as shown in Fig. 4b (main text). First, we carried out an analysis of the Stark shift for a negative V_{BG} where electrons were squeezed to a thermal Si/SiO_2 interface thus the valley splitting was negligible. Figure S3 shows the experimental Stark shift compared with the calculated result based on the standard single-valley EM theory (the parameters used are shown in the following section). Good agreement between the calculation and experimental results suggests strongly that the energy shift for a negative V_{BG} is well explained by QCSE. The lever factor β, which defines the relation between V_{BG} and F_{SOI} as $F_{SOI} = \beta V_{BG}$, is extracted from this analysis. The obtained β values are respectively 5.5 x10^5 m^{-1} for t_{SOI}=6 nm and 6.6 x10^5 m^{-1} for t_{SOI} = 4.3 nm. The fields are consistent with those estimated from the device geometries (t_{FOX} = 20 nm, t_{BOX} = 400 nm). From these results, the contribution of the valley splitting to peak shifts is extracted by comparing the energy shifts for positive and negative V_{BG} and subtracting one from the other. We thus estimated the valley splitting energies and plotted them in Fig. 4b (main text).

D. Relation between valley splitting and direct optical transition

The theory of valley splitting based on effective-mass (EM) approximation [S8-S10] was developed by Ohkawa *et al.*, where the splitting energy is given by

$$\Delta \sim \frac{\varepsilon_\Gamma}{2}\left|\int_0^\infty dz\, e^{-2ik_0 z}\left|\xi_{el}(z)\right|^2\right| \quad . \qquad \text{(S1)}$$

Here ε_Γ is the energy difference between the Γ_{15} and Γ_1^u bands, k_0 ($|k_0|$= 0.85x2.0π/a_0) is the wavenumber for the conduction band minima, and $\xi_{el}(z)$ is an electron envelope function in a quantum confinement potential. The qualitative interpretation of the equation (S1) is as follows: (i) Due to strong electron confinement, the envelope function tends to have large high frequency components. (ii) As a result, the electron wave numbers distribute around $-k_0$ and k_0 and have a significantly large component at Γ-point. (iii) Because these two valley states are both expanded by the Γ_{15} and Γ_1^u basis thus mixed at Γ-point with an energy gap of ε_Γ (see the band diagram in Fig. 1),

a_0	5.43 (Å)
k_0	$0.85(2\pi/a_0)$
m_{el}	0.98 m_0
$m_{hh <001>}$	0.28 m_0
ε_Γ	0.268 Ry

Table S1 | Constants for numerical calculation by EMA

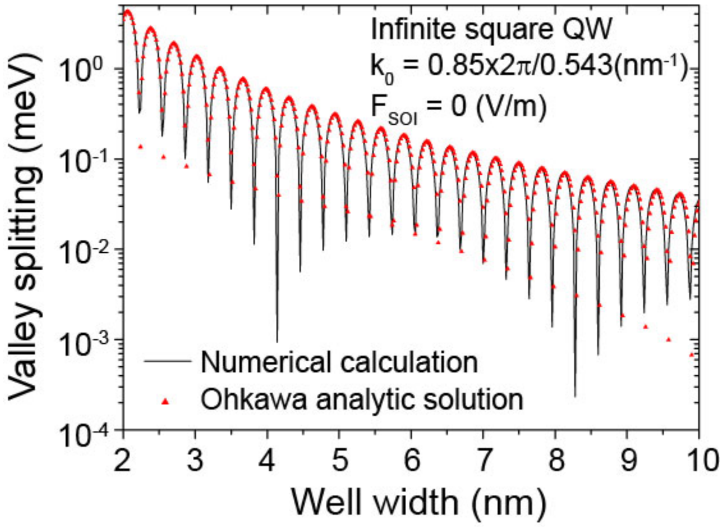

Figure S4 | Valley splitting in infinite square potential well. The solid line is given by the Fourier transform of an envelope function in infinite potential. The symbols indicate the analytic solution of valley splitting given by Ohkawa. Both results reveal oscillation with respect to t_{SOI}.

the valley splitting energy is given by ε_Γ and the $2k_0$ component of the Fourier coefficient of the squared electron envelope function.

The envelope function in eq. (S1) is obtained by solving a single-valley effective-mass equation.

$$\left(-\frac{\hbar^2}{2m^*}\frac{\partial^2}{\partial z^2}+U_0(z)-\frac{qF_{SOI}z}{\varepsilon(z)}\right)\xi(z)=E\,\xi(z) \qquad \text{(S2)}$$

where m^* is electron effective mass ($0.98m_0$), and $U_0(z)$ is a potential barrier with a value of 3.10 eV for $|z| > t_{SOI}/2$ and otherwise 0, q is the electron charge, $\varepsilon(z)$ is the dielectric constant, and F_{SOI} is the field at the SOI layer. By combining eq. (S1) with eq. (S2) we can easily estimate the valley splitting in bulk Si-MOSFETs. The constants for the following calculations are listed in Table S1. The simplest case is an infinite square well where the analytic solution of valley splitting is known [S11]. The valley splitting at a zero electric field as a function of t_{SOI} is shown in Fig. S4. For the numerical calculation, an infinite barrier is simulated by setting the $U_0(z)$ at a high value (~30 eV). In Fig. S4, the magnitude of the valley splitting oscillates as a function of well width (t_{SOI}) because of interference between $|k_0| = 0.85\text{x}2\pi/a_0$ and the minimum k step ($= 2\pi/t_{SOI}$) in the QW. Essentially,

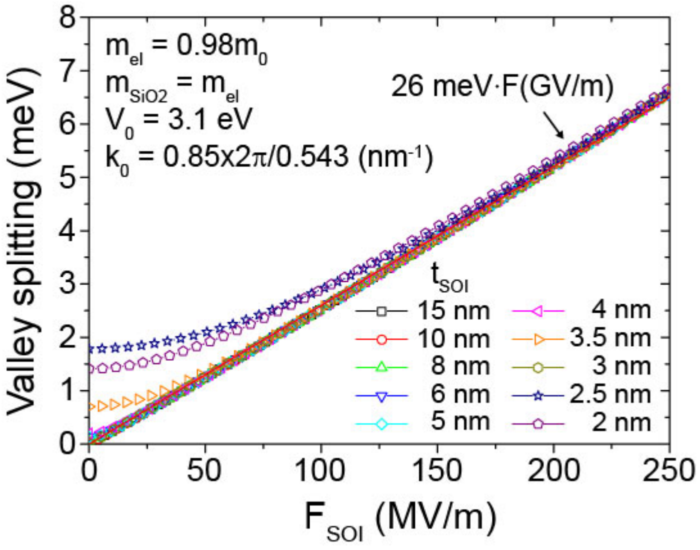

Figure S5 | Field dependence of valley splitting in finite square potential. The symbols indicate EMA in a finite potential well. For a high electric field, the coefficient of valley splitting (γ) approaches 2.6 x 10^{-11} coulomb m.

those values are inversely proportional to t_{SOI} due to an increase in the higher frequency Fourier components of the envelope function.

The field dependence of valley splitting for a finite-barrier QW with various well widths is shown in Fig. S5. For a small F_{SOI}, an analytic solution of the valley splitting gives values ranging from 0 to $\varepsilon_\Gamma \pi / t_{SOI}^3 k_0^3$ due to the interference. Even at F_{SOI} = 0, some cases have finite valley splitting Δ_0 (zero-field valley splitting) due to the interference. With increasing fields the splitting approaches a universal line and the thickness dependence disappears. This is because the electrons are likely to be confined in the triangular potential at the interface for higher field. Although there have been several reports on valley splitting using other sophisticated calculations [S12-S14], the results are basically similar to those given by eq. (S1). Therefore, we use eq. (S1) in the present work. For example, the coefficient describing the relationship between valley splitting and fields defined as $\gamma = 2\Delta/F_{SOI}$ (eV/(V/m)) is 2.6 x10^{-11} in the present calculation while it is 2.74 (±0.2) x 10^{-11} in Ref. S12. The slight deviation may be attributed to the different shape of the envelope function near the Si/SiO_2 interface, which strongly depends on the parameters used for the numerical simulation.

The use of the simple EM theory is attractive because we can easily calculate the relative EL intensities. The EL intensity of a direct optical transition (NP) is determined by Fermi's golden rule. The intensity of an inter-band transition can be described as

$$I_{NP} = \frac{2\pi}{h}\left|\left\langle u_{hh,k}\left|A\cdot p\right|u_{el,k}\right\rangle\right|^2\left|\int dz\, e^{-ik_0 z}\ \xi_{hh}(z)\xi_{el}(z)\right|^2 \delta(E_{el-hh} - E_{\hbar\omega_{photon}}) \quad \text{(S3)}$$

where the term $\delta(E_{el\text{-}hh}\text{-}E_{\hbar\omega photon})$ assures energy conservation, $u_{hh}(r)$ is the periodic part of the valence Bloch function, the $u_{el}(r)$ is that of the conduction Bloch function at the Γ-point which can be explained by the linear combination of the Γ-point basis [Γ_{25}^l, Γ_{15}, Γ_{25}^u] and [Γ_1^l , Γ_{15}, Γ_1^u] respectively, and the $A{\cdot}p$ term indicates perturbation caused by a vacuum field. Since we focus on [001] valleys, only the z component of the $A{\cdot}p$ term is taken into account in the following. The main contribution of this term is determined by momentum matrix elements $Q \equiv 2i < \Gamma_{25}^l \mid p \mid \Gamma_{15} >= 1.050\ a.u.$. As shown

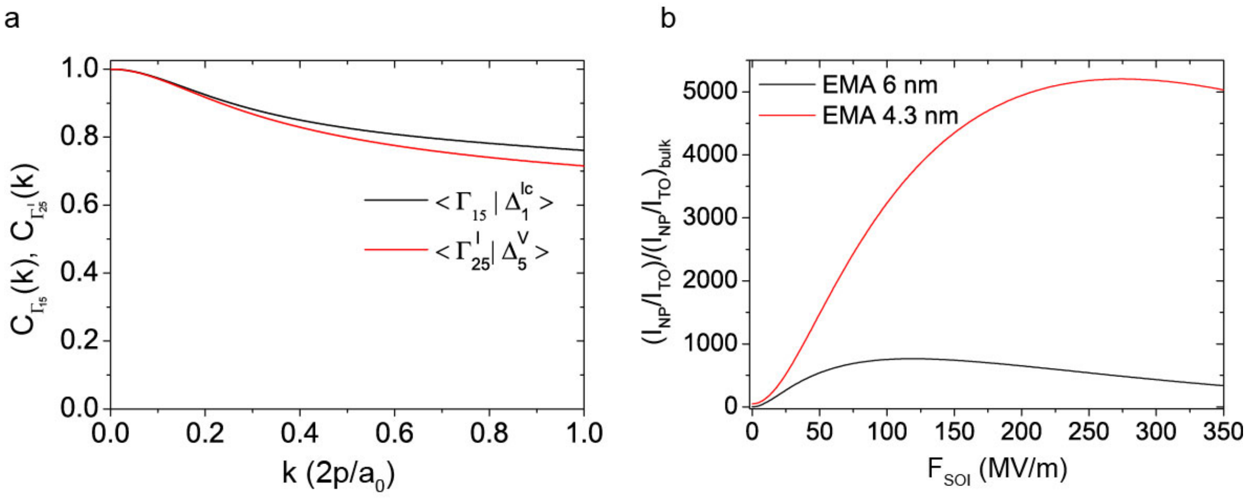

Figure S6 | Estimates of (I_{NP}/I_{TO}) / ($I_{NP}/I_{TO})_{bulk}$ by EM theory. a, Coefficients for the periodic part of Bloch function along with Γ-Δ_1^{lc}-X_1 conduction ($C_{\Gamma_{15}}(k)$) and Γ-Δ_5^{v}-X_5 valence bands ($C_{\Gamma'_{25}}(k)$). The coefficients are calculated by 15 x 15 band kp theory. **b,** The intensity ratio (I_{NP}/I_{TO}) / ($I_{NP}/I_{TO})_{bulk}$ as a function of field F_{SOI}.

in Fig. S6 (a), the coefficients $C_{\Gamma_{15}}(k)$ and $C_{\Gamma'_{25}}(k)$ change only 30% in the entire Brillouin zone after Cardona [S15] 15 x 15 band kp perturbation (We omit the details of the calculation, it is noted in Ref. S15.). According to the envelope function approximation [S16, S17], the electron wavefunction can be approximated by the product of a periodic part of the Bloch function ($u_{el,k}$ $u_{hh,k}$) and the envelope function. In eq. (S3), the modules of each component of the Bloch function are determined by the Fourier coefficient of the envelope function at given momenta. So the NP rate is essentially proportional to the dispersion of the envelope function. Therefore, we set the perturbation term for the periodic part of the Bloch function as a constant (M_{OP}) in the main text. The hole envelope function $\xi_{hh}(z)$ is obtained by solving eq. (S2) with $m^* = 0.28\ m_0$ and $U_0\ (z) = 4.8$ eV at a given field. The second term of eq. (S3) extracts the k_0 component of the Fourier coefficient for the product of heavy-hole and electron envelope functions Ξ_{k0}, which increases approximately linearly with the field due to real-space confinement. On the other hand a TO phonon mediated transition is proportional to $\left|\int dz\, \xi_{hh}(z)\xi_{el}(z)\right|^2$, which corresponds to the DC components of the Fourier coefficient for the product of the hole and electron envelope function Ξ_0. This transition decreases monotonically with the field and is commonly inversely proportional to t_{SOI} and F_{SOI}. Thus the NP/TO ratio is approximated by the following

$$I_{NP}/I_{TO} \propto \left|\langle u_{hh,k}|A_z p_z|u_{el,k}\rangle\right|^2 \left|\int dz\, e^{-ik_0 z}\, \xi_{hh}(z)\xi_{el}(z)\right|^2 \Big/ \left|\int dz\, \xi_{hh}(z)\xi_{el}(z)\right|^2 \qquad \text{(S4)}$$

In Fig. S6 (b), I_{NP}/I_{TO} compared with a bulk-like condition (NP/TO at F_{SOI} =0 and t_{SOI} =6 nm), which corresponds to the NP rate, is plotted as a function of F_{SOI}, where the perturbation term M_{OP} is constant for both thicknesses. As a rough approximation, we introduce a constant offset θ to allow us to ignore the zero-field valley splitting caused by the QW confinement, which is not observed for our case of large valley splitting, and thereby highlight the field dependence. Thus, the fitting function $f[F_{SOI}]$ can be defined by $f[F_{SOI}] = M_{op}(\Xi_{k0}[F_{SOI}] + \Delta_0)/\Xi_0[F_{SOI}] - \theta$. According to the calculation, I_{NP}/I_{TO} reaches 5,000 compared with that for F_{SOI} = 0 V/m and t_{SOI} = 6 nm (nearly bulk) conditions in the SiO_2 breakdown field (F_{SOI} ~ 300 MV/m) and it becomes large for a thinner QW. These results imply that for a high electric field the significant NP transition induced by strong

valley coupling is suppressed by the separation of the electron and hole envelope functions. Therefore, a thinner QW would be the key to maximizing the NP transition by strong valley coupling.

Supplementary references

[S1] Izumi, K., Doken, M. & Ariyoshi, H. *Electron. Lett.* **14**, 593 (1978).

[S2] Nagase, M., Ishiyama, T. & Murase, K. Proc. Int. Symp. SOI Tech. 191 (1994).

[S3] Ishiyama, T. & Nagase, M. *Jpn. J. Appl. Phys.*, Part 1, **34**, 6019 (1995).

[S4] Ishiyama, T. Nagase, M. & Omura, Y. Evolution of step-terrace structure at Si-SiO_2 interface in SIMOX substrate during annealing. *Appl. Surf. Sci.* **190**, 16 (2002).

[S5] Noborisaka, J. *et al.* Strong Stark effect in electroluminescence from phosphorous-doped silicon-on-insulator metal-oxide-semiconductor field-effect transistors. *Appl. Phys. Lett.* **98**, 033503 (2011).

[S6] Dean, P. J. & Haynes, J. R. & Flood, W. F. New radiative recombination processes involving neutral donors and acceptors in silicon and germanium. *Phys. Rev.* **161**, 711 (1967).

[S7] Miller *et al.,* Band-edge electroabsorption in quantum well structures: The Quantum-Confined Stark Effect. *Phys. Rev. Lett.* **53**, 2173 (1984)

[S8] Ohkawa, F. J., & Uemura, Y. Theory of valley splitting in an N-channel (100) inversion layer of Si. *J. Phys. Soc. Japan.* **43**, 907 (1977).

[S9] Ohkawa, F. J., & Uemura, Y. Theory of valley splitting in an N-channel (100) inversion layer of Si. *J. Phys. Soc. Japan.* **43**, 917 (1977).

[S10] Ohkawa, F. J., & Uemura, Y. Theory of valley splitting in an N-channel (100) inversion layer of Si. *J. Phys. Soc. Japan.* **43**, 925 (1977).

[S11] Ohkawa, F. J. Electric break-through in an inversion layer: exactly solvable model. Sol. Stat. comm. **26**, 69 (1978).

[S12]. Saraiva, A. L. *et al.* Intervalley coupling for interface-bound electrons in silicon: An effective mass study. *Phys. Rev. B* **84**, 155320 (2011).

[S13] Boykin, T. B. *et al.* Valley splitting in strained silicon quantum wells. *Appl. Phys. Lett.* **84**, 115 (2004).

[S14] Boykin, T. B. *et al.* Valley splitting in low-density quantum-confined heterostructures studied using tight-binding models. *Phys. Rev. B* **70**, 165325 (2004).

[S15] Cardona. M. & Pollak. F. H. Energy-band structure of germanium and silicon: The kp method. *Phys. Rev.* **142**, 530 (1966).

[S16] Luttinger, J. M. & Kohn, W. Motion of electrons and holes in perturbed periodic fields. *Phys. Rev.* **97**, 869 (1955)

[S17] Basterd, G. Superlattice band structure in the envelope function approximation. *Phys. Rev. B.* **24**, 5693 (1981).
